# Functional toll-like receptor 4 links endotoxin sensing to platelet priming in feline platelets

**DOI:** 10.3389/fvets.2025.1731802

**Published:** 2026-01-07

**Authors:** Ronald H. L. Li, Meg Shaverdian, Cheyenne Chen, Claire Stuhlmann, Joshua A. Stern, Nghi Nguyen

**Affiliations:** 1 Department of Clinical Sciences, College of Veterinary Medicine, North Carolina State University, Raleigh, NC, United States; 2 Feline Health Center, College of Veterinary Medicine, North Carolina State University, Raleigh, NC, United States; 3 Department of Surgical and Radiological Sciences, School of Veterinary Medicine, University of California, Davis, Davis, CA, United States

**Keywords:** antiplatelet therapy, arterial thromboembolism, hypertrophic cardiomyopathy, immunothrombosis, sepsis

## Abstract

**Objective:**

To characterize toll-like receptor 4 (TLR4) expression in feline platelets and to assess the priming potential of *Escherichia coli* lipopolysaccharide (LPS) in the presence or absence of physiologic agonists. In addition, the downstream effects of TLR4 activation on arachidonic acid (AA)-mediated signaling were investigated.

**Methods:**

Eighteen healthy staff- and student-owned cats were enrolled. Washed platelets prepared from whole blood were analyzed for total and surface TLR4 expression using Western blotting, flow cytometry, and super-resolution immunofluorescence microscopy in the presence or absence of stimulation. The priming potential of LPS was evaluated by measuring alpha-granule secretion by P-selectin expression and thromboxane B_2_ (TxB_2_) generation, as a surrogate of TxA_2_, in response to adenosine diphosphate (ADP) or AA using flow cytometry and ELISA, respectively. To further examine TLR4-dependent signaling, phosphorylation of vasodilator-stimulated phosphoprotein (P-VASP) was quantified following stimulation with AA and LPS from *Rhodobacter sphaeroides* (LPS-RS).

**Results:**

Thrombin stimulation significantly upregulated both surface and total platelet TLR4 expression. While LPS alone did not induce α-granule secretion with or without ADP, it reversed the inhibitory effect of AA by enhancing P-selectin expression and potentiating TxB_2_ generation. This priming effect of LPS was mediated through TLR4, resulting in decreased cytoplasmic P-VASP, a marker associated with platelet inhibition.

**Discussion:**

This study is the first to demonstrate functional TLR4 expression in feline platelets. Activation of TLR4 sensitizes platelets to AA by augmenting TxA_2_ production and attenuating prostaglandin-dependent inhibitory pathways. These findings highlight a novel mechanism by which platelet TLR4 contributes to immunothrombosis and may promote thrombotic risk in cats.

## Introduction

Cardiogenic arterial thromboembolism (CATE) is a devastating complication in cats with cardiomyopathies, associated with an in-hospital mortality rate of approximately 50% and a recurrence rate of 15–75% (1–3). CATE develops when an intracardiac thrombus embolizes to the distal arteries, resulting in partial or complete vascular occlusion. The ensuing tissue ischemia can trigger ischemic reperfusion injury, systemic inflammation, and multi-organ dysfunction (4). Although the pathogenesis of CATE is not well understood, it likely involves all three components of Virchow's triad: systemic hypercoagulability, endothelial injuries and blood stasis. Platelets, as primary effectors of hemostasis, play a central role in the process. Increased platelet activation and platelet reactivity have been demonstrated in cats with cardiomyopathies including hypertrophic cardiomyopathy (HCM) and transient myocardial thickening following wildfire-related injuries (5–7). Beyond their hemostatic role, platelets are increasingly recognized as innate immune cells capable of responding to systemic infection and inflammation, and mediating immunothrombosis (8). Immunothrombosis, a vital component of innate immunity, promotes localized and controlled microvascular thrombosis to contain pathogens and promote tissue repair (9). However, dysregulated immunothrombosis in cardiovascular diseases and systemic inflammation, such as sepsis, severe burns and ischemic reperfusion injury, can precipitate pathologic thrombosis, impair oxygen delivery, and contribute to organ failure and death (10–13).

Platelet priming, which reduces the activation threshold of platelets, causing unrestrained platelet activation, often precedes platelet-endothelial and platelet-leukocyte interactions. These interactions are essential for immunothrombosis and, in particular, for the formation of neutrophil extracellular traps (NETs), web-like cell-free DNA decorated with histones and granular proteins (14). Overzealous production of NETs, reported in cats with HCM and CATE, may drive systemic hypercoagulability and thromboembolic complications (15). We previously observed that a substantial proportion of cats rescued and treated for wildfire-related injuries and smoke inhalation developed transient myocardial thickening and intracardiac thrombosis (16). These cats not only exhibited increased circulating activated platelets but, in cases with active thrombosis, also demonstrated an exaggerated response to the platelet agonist, arachidonic acid (AA). In contrast, platelets from healthy cats exposed to the same concentration of AA showed marked inhibition, likely due to increased endogenous prostaglandins (7). These findings suggest that platelet priming in cats with systemic inflammation or cardiovascular disease, might be mediated by enhanced thromboxane A_2_ (TxA_2_) production or modulation of the intrinsic inhibitory pathways mediated by prostaglandin-dependent inhibitory pathways.

Recent studies indicate that canine platelets express functional toll-like receptor 4 (TLR4), enabling them to respond to lipopolysaccharides (LPS) from Gram-negative bacteria and endogenous alarmins such as high mobility group box 1 released during inflammation (17, 18). Activation of platelet TLR4 initiates complex intracellular signaling cascades that lead to proinflammatory cytokine production and further platelet activation. In both humans and dogs, TLR4-mediated platelet activation is partially dependent on the arachidonic acid pathway, wherein membrane-derived AA is converted to TxA_2_, a positive feedback mediator that amplifies platelet activation (17, 19, 20).

It remains unclear whether the heightened platelet response to AA and increased thrombosis observed in cats with HCM and wildfire-related injuries are linked to the upregulation of platelet TLR4. To date, neither TLR4 expression nor its function has been characterized in feline platelets. Understanding TLR4-mediated signaling in cats could provide critical insights into platelet-driven inflammatory and thrombotic mechanisms and inform targeted therapies for inflammatory conditions such as sepsis, burn injuries, and systemic inflammatory response syndrome. We therefore hypothesized that feline platelets express functional TLR4, enabling them to be primed by *Escherichia coli (E.coli)* LPS via TxA_2_ overproduction and inhibition of the AA-induced P-VASP-pathway. To test this hypothesis, we pursued the following objectives: (1) To characterize platelet TLR4 expression in the presence or absence of thrombin; (2) To assess the priming potential of *E.coli* LPS in the presence or absence of adenosine diphosphate (ADP) or AA by measuring P-selectin surface expression and thromboxane B_2_ (TxB_2_) secretion; (3) To evaluate the role of TLR4 in AA-mediated signaling by quantifying VASP phosphorylation and TxB_2_.

## Methods

### Animals

Eighteen clinically healthy staff- or student-owned cats older than 1 year of age were enrolled for this study. All cats received a physical examination, complete blood count, biochemical profile, and an echocardiogram. Echocardiograms were performed as previously described (21). Cats were excluded if they were diagnosed with hypertrophic cardiomyopathy or other forms of cardiomyopathy on echocardiogram, on any concurrent medications (except gabapentin for sedation) or had any abnormalities in blood work. The animal studies were approved by University of California, Davis Institutional Animal Care and Use Committee (Protocol # 21037). The studies were conducted in accordance with the local legislation and institutional requirements. Written informed consent was obtained from the owners for the participation of their animals in this study.

### Generation of platelet rich plasma and washed platelets

Approximately 4 ml of whole blood was collected from the medial saphenous using a 21- or 23-gauge butterfly needle after written consent was obtained. Whole blood was immediately aliquoted to tubes containing 3.2% sodium citrate, then gently inverted and carefully inspected for clots. To facilitate erythrocyte sedimentation, blood tubes were placed vertically in a 37 °C bead bath for 30 min. Platelet rich plasma (PRP) was generated by centrifugation at 200 × G for 5 min (no brakes) at room temperature. Platelet swirling was assessed to test the quality of PRP (resting state), and platelet count was obtained using an automated analyzer (HM5, Abaxis). Following centrifugation, platelets were rested in a 37 °C bead bath for an additional 30 min before proceeding with further experiments. Due to the limited amount of blood collected from each cat, not all enrolled cats had enough platelets for the assays described below.

### Toll-like receptor 4 detection by flow cytometry, Western blot analysis and immunofluorescence microscopy

Platelets from 6 cats were isolated to characterize TLR4 expression. Total cellular TLR4 expression was semi-quantitatively measured by Western blot analysis. Plasma proteins were separated from PRP by gel-filtration over a Sephrose 2B column eluted in pre-warmed Tyrodes HEPES buffer (pH 7.2, 5 mM dextrose, without divalent cations). Gel-filtered platelets were then adjusted to 1 × 10^8^ cells/ml and were either unstimulated (resting) or stimulated with 0.1 U/ml bovine α-thrombin (Protylix, Esses Junction, VT) for 15 min at 37 °C before being lysed in 1 × Laemmli buffer containing 355 mM 2-mercaptoethanol (Bio-Rad, Hercules, CA), boiled (5 min), placed on ice (5 min), loaded onto 4–15% polyacrylamide gels (Bio-Rad, Hercules, CA) and separated by sodium dodecyl sulfate-polyacrylamide gel electrophoresis. Proteins were then transferred to polyvinylidene difluoride membranes and confirmed by Ponceau S staining (Thermo Fisher Scientific, Waltham MA). After blocking with 10% bovine serum albumin for 3 hours at room temperature, membranes were incubated in rabbit polyclonal antibody to TLR4 (1:4,000, ab13556, Abcam, Cambridge, MA), previously shown to cross-react with feline TLR4, overnight at 4 °C (22). This was followed by incubation with secondary goat anti-rabbit IgG conjugated to horse radish peroxidase (1:20,000, ab6013, Abcam, Cambridge, MA) for 1 hour at room temperature. Chemiluminescent substrate (WesternBright Quantum, Advansta, San Jose, CA) was added directly on the blots and imaged (Fluorchem E, Protein Simple, San Jose, CA). Beta-actin, used as loading control, was probed using mouse monoclonal antibody (1:4,000, ab8226, Abcam, Cambridge, MA), followed by secondary goat anti-mouse IgG conjugated to horse radish peroxidase (1:20,000, ab97023, Abcam). Western blots were analyzed by available software (FIJI, NIH)

To detect surface TLR4 by flow cytometry, PRP standardized to 1 × 10^7^ cells/ml with Tyrodes-HEPES was incubated with fluorescein isothiocyanate (FITC)-conjugated rabbit polyclonal antibody to TLR4 (1:100, ab13556, Abcam, Cambridge, MA) in the presence or absence of thrombin. After 45 min, platelets were fixed in 1% methanol-free paraformaldehyde and analyzed by flow cytometry.

Additionally, surface TLR4 was assessed by immunofluorescence microscopy. Gel-filtered platelets (1 × 10^6^/ml), treated with 10 mM PGE_1_ (Millipore sigma), were allowed to adhere onto poly-D-lysine coverslips which were then fixed in 1% paraformaldehyde for 30 min at 37 °C, and air dried for 5 min. After washing in phosphate buffered saline, platelets were first blocked with 5% goat serum (2 h, room temperature), and subsequently incubated with biotinylated rabbit anti-human polyclonal antibody to TLR4 (1:5, ab13556, Abcam) overnight at 4 °C. After washing, platelets were incubated in streptavidin conjugated to Alexa Fluor 555 (1:25 in 5% goat serum, 1 h at 37 °C). Additionally, integrin β3a (CD61) was detected using mouse anti-human CD61 monoclonal antibody (1:100 in 5% goat serum, overnight at 4 °C), followed by incubation with Alexa Fluor 405-conjugated goat anti-mouse IgG (1:50, eBioscience, ThermoFisher Scientific, Waltham, MA). After washing, coverslips were mounted onto glass slides using anti-fade mounting medium, and cured overnight. Fluorescent images were acquired as previously described using confocal as well as super-resolution stimulated emission depletion (STED) microscopy (Leica TCS SP8 STED 3x, Leica Microsystems, Buffalo Grove, IL). To avoid photobleaching, CD61 was first detected by confocal microscopy excited with 405 nm with 1–6 nsec HyD gating. Alexa Fluor 555 (TLR4) was then excited with 555 and 660 nm STED depletion laser. Imaging powers of STED wavelengths were set to 20 to 50% of excitation wavelengths.

### Analysis of platelet P-selectin expression

Platelets from 8 cats were isolated and standardized to 1 × 10^7^ cells/ml with Tyrodes-HEPES (5 mM dextrose, pH 7.2, no divalent cations) before treatment with 0, 10, 100, 1000, and 5000 ng/mL *E.coli* O55:B5 LPS (InvivoGen, San Diego, CA) in the presence or absence of 2.5 μM ADP (MilliporeSigma, Burlington, MA) or 1 mM AA (Chronolog, Havertown, PA) (15 min, 37 °C). Unstimulated (resting) platelets and platelets treated with 10 μM ADP served as negative and positive controls, respectively. Following activation, platelets were incubated with FITC-conjugated rat anti-mouse monoclonal antibody to P-selectin (CD62P) (1:200, BD Biosciences, San Diego, CA). Platelets were identified by mouse anti-human monoclonal antibody to platelet integrin, β3a (CD61) conjugated to allophycocyanin (APC) (1:500, eBioscience, San Deigo, CA) for 45 min at 37 °C. Platelets were then fixed in 0.1% paraformaldehyde and analyzed using a 5-color flow cytometer (Beckman-Coulter FC500, Beckman-Coulter Inc., Miami, FL).

### Thromboxane B_2_ ELISA

To determine if LPS-mediated priming is dependent on the COX pathway, we measured TxB_2_ in platelet supernatant in 7 cats. First, PRP was standardized to 1 × 10^8^ platelets/ml with Tyrodes-HEPES (5 mM dextrose, pH 7.2, no divalent cations) before treatment with 1,000 ng/ml LPS and 1 mM AA (Chronolog) for 15 min at 37 °C. Platelets treated with 100 μM acetylsalicylic acid (ASA) (Sigma-Aldrich, St. Louis, MO) (30 min, 37 °C) served as negative control. Platelet supernatant, generated by centrifugation at 1,500 rpm for 15 min at room temperature, was collected, flash frozen in liquid nitrogen and stored at −80 °C until analysis using a commercially available ELISA kit (Enzo Life Sciences Inc., Farmingdale, NY).

### Detection of intracellular vasodilator-stimulated phosphoprotein phosphorylation

To determine if platelet priming by LPS was mediated by modulation of the inhibitory signal generated by PGE_1_, we measured phosphorylation of cytoplasmic vasodilator-stimulated phosphoprotein (P-VASP) as previously described (23). First PRP (2 × 10^7^/ml) was simultaneously treated with 1,000 ng/ml *E. coli* LPS and 10 μM PGE_1_ (MilliporeSigma, Burlington, MA), for 15 min at 37 °C. Platelets were first treated with 10 μM PGE_1_ or 10 μM ADP served as positive and negative controls, respectively. Cells were fixed immediately with 1% methanol-free paraformaldehyde (Thermo Fisher, Waltham, MA) prior to brief permeabilization using 0.25% detergent (NP-40 Surface-AMPs Detergent Solution, Thermo Fisher, Waltham, MA). Permeabilized platelets were immediately washed and resuspended in Tyrodes HEPES before labeling with mouse polyclonal antibody conjugated to FITC that detects phosphorylation of VASP at serine 239 (5μg/ml, ALX-804-240F-C100, Enzo Life Sciences, Farmingdale, NY). Platelets were analyzed by flow cytometry immediately.

### Platelet TLR4 inhibition

To determine if LPS-mediated platelet priming is dependent on TLR4, platelets from 6 cats were pre-treated with 50 μg/ml ultrapure lipopolysaccharides from *Rhodobacter sphaeroides* (LPS-RS) (InvivoGen, San Diego, CA), a specific TLR4 antagonist, or the same volume of vehicle control (sterile water) for 1 h at 37 °C.

### Flow cytometry analysis

Platelets were identified by forward and side scatter properties using 0.9 μm and 3 μm calibration beads as previously described (6). Additionally, the platelet gate was established as CD61-positive events by fluorescence-minus-one and isotype controls. Compensation for fluorescence spectral spillover was applied using anti-mouse IgG isotype controls conjugated to experimental fluorophores, FITC and APC with positive and negative mouse compensation beads (BD Biosciences, San Diego, CA) in identical experimental conditions. Compensation matrixes and flow cytometry data were calculated and analyzed using commercially available software (FlowJo, Tree Str Inc., Ashland, OR). Upregulation in P-selectin expression was measured as fold change in median fluorescence intensity (MFI) between activated platelets and resting platelets using the following equation:


$$\text{Response to agonist }=\text{Log 10 }(\frac{\text{MFI activated}}{\text{MFI resting}})$$ (1)

### Statistical analysis

Based on preliminary data, a sample size calculation determined that a minimum of six cats was required to detect a 30% difference in platelet activation with 80% power and an alpha-priori of 0.05. Due to the likelihood of *in vitro* platelet activation and spontaneous aggregation, a total of eight cats were needed for each objective. Data normality was assessed using the D'Agostino and Pearson tests. Normally distributed data were presented as mean ± standard deviation, while non-parametric data were reported as median and interquartile range (IQR). Paired data were compared using paired *t*-tests and Wilcoxon signed-rank tests, as appropriate. Repeated measures ANOVA or its non-parametric equivalent, Kruskal–Wallis's test, was used to evaluate treatment effects, followed by Tukey's *post-hoc* test for multiple comparisons. Statistical significance was defined as *p* < 0.05. All analyses were performed using GraphPad Prism version 10.0 (GraphPad Software, La Jolla, CA).

## Results

### Animals

The mean age of the cats was 3 years ± 2.4. All cats were Domestic Shorthair cats. Of the 20 cats, 8 were female spayed and 12 were male neutered. One cat had left ventricular cavity obliteration and one cat was found to have dynamic left ventricular outflow tract obstruction. Those two cats were excluded from the study. The remaining 18 cats had normal echocardiograms.

### Feline platelets express TLR4, which is upregulated in the presence of thrombin

**Figure 1:**
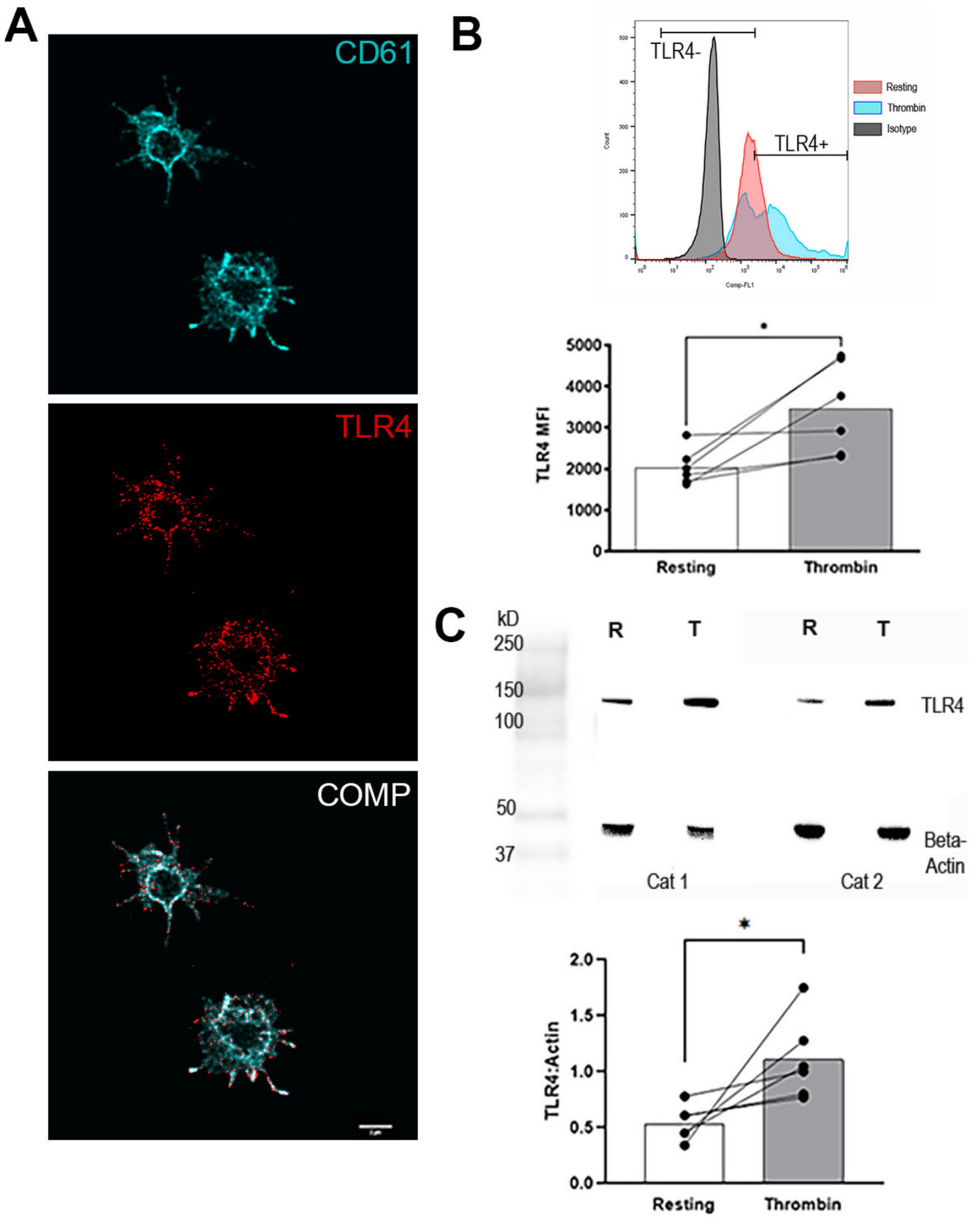
Feline platelets express Toll-like receptors 4 (TLR4). **(A)** Representative confocal and super resolution immunofluorescence microscopy images of unpermeabilized resting platelets stained for the integrin β3 (cyan) and TLR4 (red). Scale bar = 2 μm. **(B)** Representative histograms demonstrating TLR4 surface expression in isotype control (gray), resting platelets (red) and thrombin-activated platelets (blue) in a cat by flow cytometry. Before-and-after dot plots demonstrating surface TLR4 expression in resting and thrombin-stimulated platelets from 6 cats. TLR4 surface density, measured as median fluorescence intensity (MFI), was significantly increased with thrombin stimulation. **(C)** Representative immunoblots of TLR4 (at ~150 kDa) and the loading control, beta-actin (42kDa), in platelet lysates from 2 cats, illustrating the upregulation of the total TLR4 expression after thrombin stimulation. Thrombin significantly upregulated platelet TLR4 expression as assessed by densitometry of Western blots relative to the loading control. ^*^p < 0.05. Bars represent mean.

Consistent with these flow cytometry findings, confocal and super-resolution microscopy demonstrated that TLR4 is distributed uniformly across the membrane of resting feline platelets (Figure 1A). The number of platelets expressing surface TLR4 in unstimulated (resting) platelets (87.2% ± 7.9) did not differ from thrombin-activated platelets (83.6% ± 5.7, *p* = 0.29). However, overall surface density increased significantly after thrombin stimulation (MFI_resting_ = 2039 ± 439.6 vs. MFI_thrombin_ = 3459 ± 1107, *p* = 0.03) (Figure 1B). Similarly, the total cellular expression of TLR4 based on Western blot analysis demonstrated significant upregulation in thrombin-activated platelets (TLR4:Actin = 0.5 ± 0.2) compared to resting platelets (TLR4:Actin = 1.1 ± 0.4, *p* = 0.036) (Figure 1C).

### Lipopolysaccharides do not induce alpha-granule secretion, but reverse arachidonic acid-mediated inhibition

**Figure 2:**
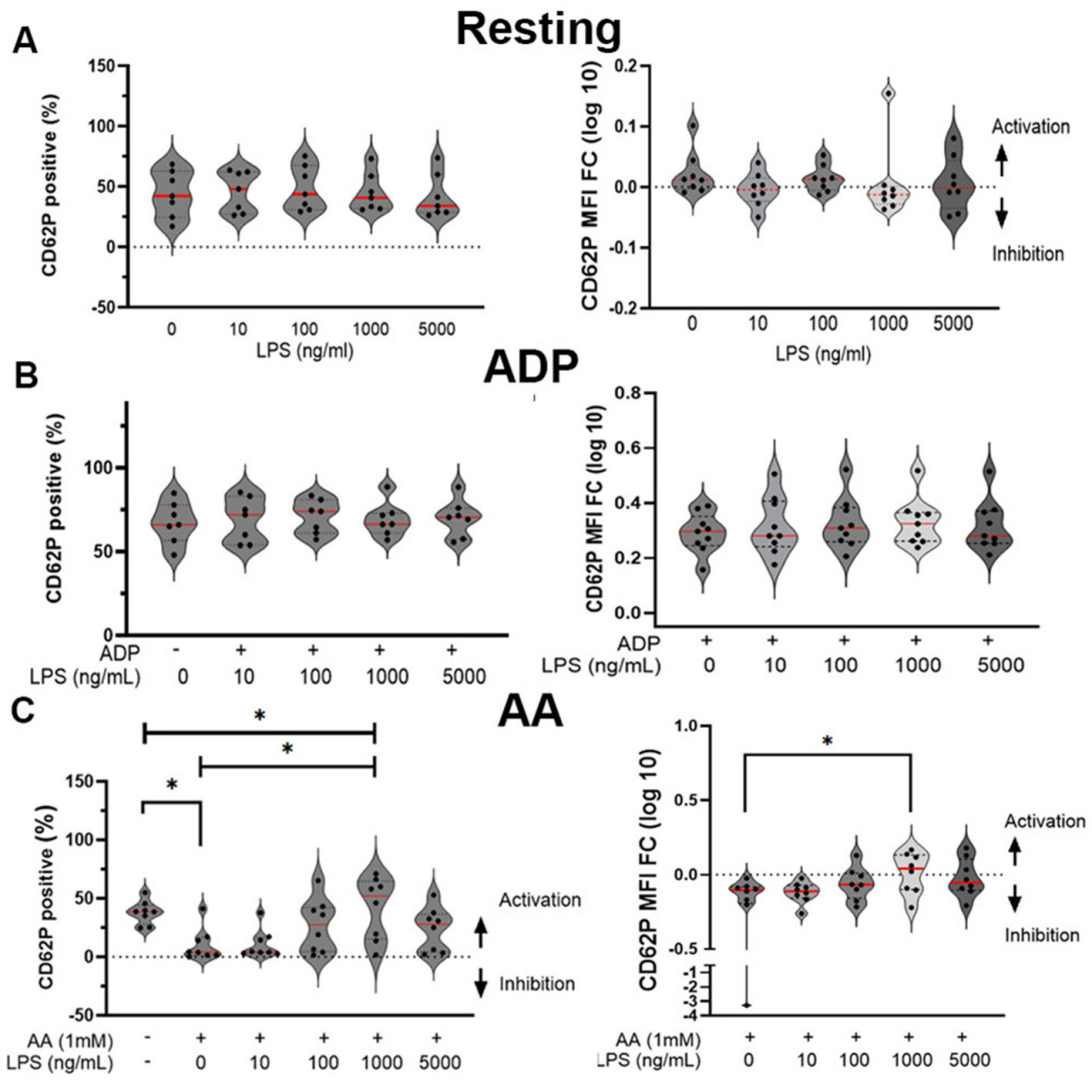
Lipopolysaccharide (LPS) alone did not induce alpha-granule secretion but restored the activating potential of arachidonic acid in feline platelets. **(A)** Violin plots showing the percentage of CD62P-positive (P-selectin) platelets (left) and log-transformed fold change in median fluorescence intensity (MFI FC) (right) after stimulation with increasing concentrations of LPS (0, 10, 100, 1,000 and 5,000 ng/mL). LPS alone did not significantly alter surface P-selectin expression. For MFI FC calculations, all treatments including vehicle control (the 0 ng/mL LPS) were measured against resting platelets (baseline). **(B)** Violin plots illustrating the effect of LPS pre-treatment on ADP-induced platelet activation, as assessed by number of CD62P-positive platelets (left) and CD62P MFI fold change (log 10) (right). LPS did not significantly alter ADP-induced P-selectin expression. **(C)** Violin plots demonstrating the priming effect of LPS on arachidonic acid (AA)-treated platelets. AA alone markedly inhibited P-selectin expression, and co-incubation with LPS significantly reversed this inhibitory effect in a dose-dependent manner by not only increasing the number of CD62P-positive platelets (left) but also potentiating reactivity to AA (right). LPS at 1,000 ng/ml restored AA-induced platelet activation. ^*^p < 0.05. Each dot represents an individual animal (*n* = 8). Red line represents the median.

Increasing concentrations of *E.coli* LPS did not elicit a dose response in increasing alpha-granule secretion, measured by the number of P-selectin positive cells (*p* = 0.47) and upregulation of surface P-selectin (*p* = 0.53) (Figure 2A). Similarly, LPS failed to upregulate ADP-mediated P-selection expression among all concentrations [CD62P positive (%) *p* = 0.55; CD62P MFI FC *p* = 0.28] (Figure 2B). As expected, AA inhibited alpha-granule secretion by decreasing the number of P-selectin-positive platelets (AA = 4.1% IQR: 1.6 to 16.5 vs. Resting = 38.7% IQR: 27.8 to 44.1, *p* = 0.04) and downregulating surface density (MFI FC_AA_ = −0.1 IQR: −0.2 to −0.08). However, co-treatment of platelets with LPS reversed AA-mediated inhibition in a dose-dependent manner (*p* = 0.032). Compared to platelets treated with AA alone, LPS at a concentration of 1,000 ng/ml significantly increased the number of P-selectin positive platelets (52.1% IQR: 15.2 to 64.8, *p* = 0.008) and augmented the activating response to AA (MFI FC_AA+LPS_ = 0.04, IQR: −0.010 to 0.13 vs. MFI FC_AA+VC_ = −0.10, IQR: −0.19 to −0.080) (*p* = 0.036) (Figure 2C).

### Lipopolysaccharide potentiates thromboxane B_2_ generation by arachidonic acid

**Figure 3:**
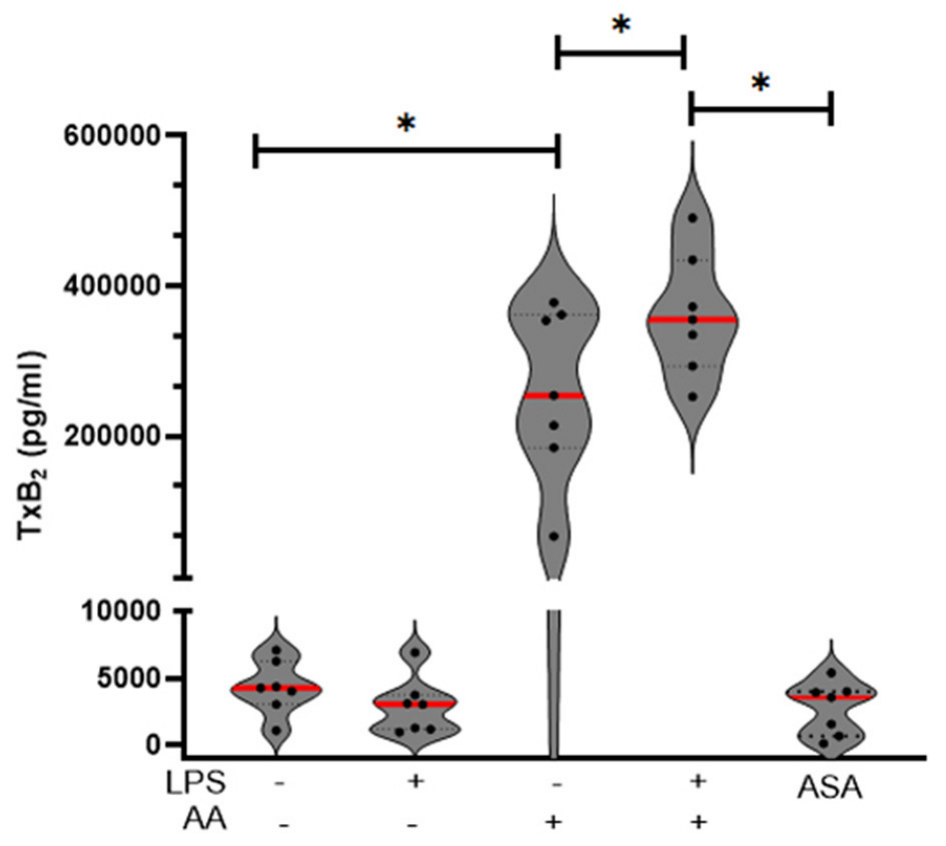
LPS potentiated AA-induced thromboxane B_2_ production and modulates cAMP signaling in feline platelets. Violin plots showing thromboxane B_2_ (TxB_2_) concentrations in feline platelets under resting conditions or following stimulation with arachidonic acid (AA), with or without LPS co-treatment. AA alone markedly increased TxB_2_ production, and the addition of LPS further potentiated this effect. LPS alone did not significantly alter TxB_2_ levels. Acetylsalicylic acid (ASA)-treated platelets served as negative control. ^*^p < 0.05.

Next, we assessed if LPS could enhance AA-induced thromboxane A_2_ (TxA_2_) generation by measuring TxB_2_, a stable metabolite of TxA_2_, in platelet supernatant. As expected, LPS alone did not induce TxB_2_ generation (LPS = 3,061 pg/ml IQR: 1,201–3,746 vs. Resting = 4,288 pg/ml IQR: 3,066–6,284, *p* = 0.08). Treatment with exogenous AA induced a significant increase in TxB_2_ generation (AA = 254,278 pg/ml IQR: 184,769–361,607) compared to resting platelets (*p* = 0.001). This response was further potentiated in the presence of LPS (AA + LPS = 355,441 pg/ml IQR: 293,405 to 434,645; *p* = 0.03) (Figure 3).

### Lipopolysaccharide decreases the inhibitory signal generated by arachidonic acid via platelet toll-like receptor 4

**Figure 4:**
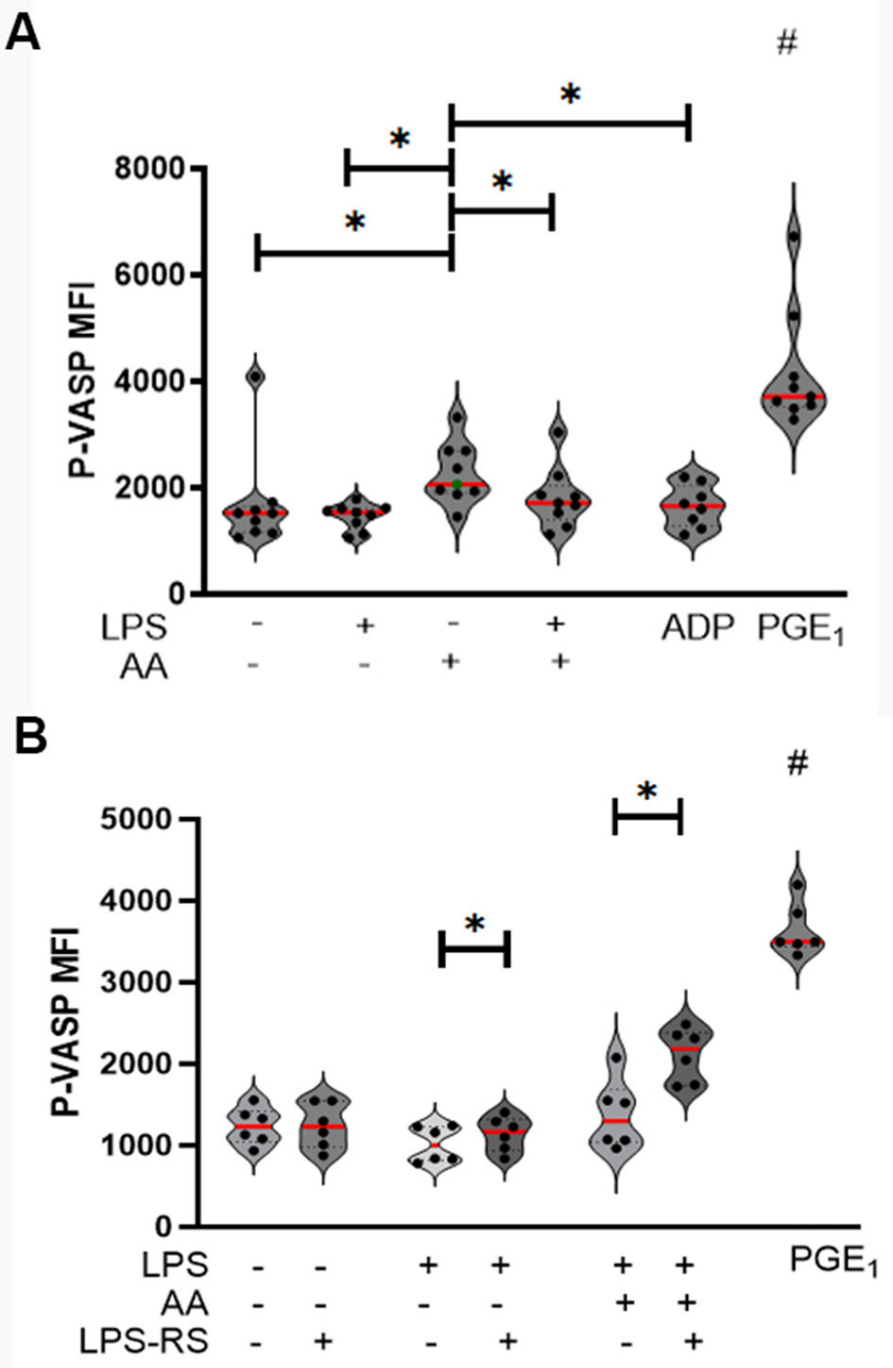
Violin plots of phosphorylated vasodilator-stimulated phosphoprotein (P-VASP) median fluorescence intensity (MFI), used as a marker for intracellular cyclic AMP (cAMP) signaling. **(A)** AA significantly elevated P-VASP expression. Co-treatment with LPS and AA significantly reduced P-VASP levels, suggesting a reversal of AA-induced platelet inhibition. ADP, which significantly decreased P-VASP compared to AA-treated platelets, served as negative control (*n* = 9). **(B)** TLR4 inhibition by treatment with LPS from *Rhodobacter sphaeroides* (LPS-RS) not only reversed LPS-mediated decrease in P-VASP, it also restored AA-mediated inhibition by increasing intracellular P-VASP (*n* = 6). PGE_1_-stimulated platelets are shown as positive control for cAMP activation. ^*^p < 0.05, # indicates significant difference from all other conditions. Each dot represents an individual animal. Bars represent the median.

Given that AA is a precursor of platelet inhibitors like prostacyclin and prostaglandin, we assessed intracellular P-VASP, a marker of platelet inhibition, in AA-treated platelets (*n* = 9) in the presence or absence of LPS. As expected, AA significantly increased P-VASP compared to resting platelets (MFI_rest_ = 1,522 IQR: 1,163–1,655 vs. MFI_AA_ = 2,063 IQR: 1,906–2,696, *p* = 0.04). While LPS alone did not affect P-VASP (MFI_LPS_ = 1,537 IQR: 1,240–1,618 vs. MFI_rest_, *p* = 0.6), LPS modulated the inhibitory effect of AA by decreasing P-VASP (MFI_AA+LPS_ = 1,719 IQR: 1,398–2,044 vs. MFI_AA_ = 2,063 IQR: 1,906–2,696, *p* = 0.04). PGE_1_-treated platelets, which served as positive controls, had significantly higher P-VASP (MFI_PGE1_ = 3,718 IQR: 3,523–4,661) compared to other conditions (*p* < 0.01) (Figure 4A).

To determine if TLR4 inhibition with LPS-RS restored intracellular P-VASP, we measured platelet P-VASP in untreated, LPS, and AA-treated platelets in the presence or absence of LPS-RS (*n* = 6). TLR4 inhibition increased P-VASP in LPS-treated platelets (MFI_LPS − RS_ = 1,014 ± 216.8 vs. MFI_LPS_ = 1,140 ± 212.0, *p* = 0.04) to a limited extent. However, marked elevation in P-VASP was detected with TLR4 inhibition in AA and LPS-treated platelets (MFI_LPS − RS+AA+LPS_ = 2,111 ± 325.9 vs. MFI_AA+LPS_ = 1,375 ± 424.8, *p* = 0.01), indicating a shift back to inhibitory signal generated by prostaglandins (Figure 4B).

## Discussion

**Figure 5:**
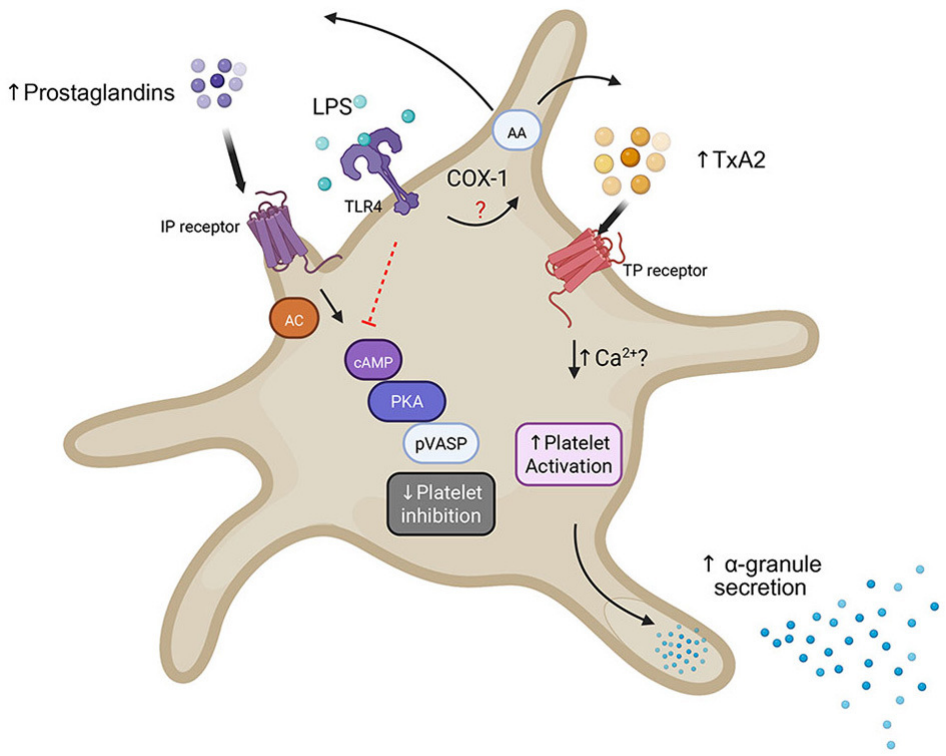
Schematic diagram illustrating potential mechanisms of platelet priming by TLR4 in feline platelets. Downstream signaling of TLR4 elicited by LPS binding leads to increased thromboxane A_2_ (TxA_2_) generation likely due to increased activation of platelet cyclooxygenase-1 (COX-1). At the same time, TLR4 downstream signaling decreases platelet inhibition mediated by prostaglandins via prostacyclin receptor (IP) by decreasing cyclic amino monophosphate (cAMP), protein kinase A (PKA) and phosphorylation of vasodilator-stimulated phosphoprotein (P-VASP), resulting in platelet priming and increased alpha-granule secretion.

In this study, we demonstrated for the first time that feline platelets express functional TLR4, which is further upregulated upon thrombin stimulation. We also showed that feline platelets respond to LPS through TLR4 activation, eliciting a priming effect via novel non-genomic pathways. This not only enhances thromboxane A_2_ secretion, but also shifts the AA-mediated inhibitory signal to an activating one, to potentiate platelet activation (Figure 5). Together, these findings support the hypothesis that feline platelets play an active role in immunothrombosis by directly responding to endotoxins and potentially promoting thrombosis via platelet priming.

Feline platelets express constitutive level of TLR4, consistent with its highly conserved role in pathogen-associated molecular pattern recognition across mammalian platelets. Similar to canine platelets, thrombin stimulation upregulates platelet TLR4, likely through caplain-myosin-9 mediated translocation of TLR4 from alpha-granules to the platelet membrane (17, 24). Western blot analysis of platelet lysates further demonstrates that thrombin activation promotes *de novo* synthesis of TLR4. Although the precise mechanism remains unclear, TLR4 mRNA modification through N6-methyladenosine (m6A) methylation in other immune cells like neutrophils enhances translation, limits degradation and, thereby, upregulates under systemic inflammation. This suggests that during systemic inflammation and hypercoagulability, increased thrombin generation could augment the immune and hemostatic capacity of feline platelets. Supporting this concept, a recent single-cell transcriptomic study in septic human patients showed that platelets undergo m6a methylation to enhance its immune function in response to systemic inflammation (25). Whether comparable TLR4 upregulation occurs in cats with naturally occurring systemic inflammation remains to be determined.

Our previous observational study showed that prothrombotic cats with wildfire related injuries had persistent platelet activation in response to AA. Compared to those without clinical evidence of thrombosis, increased platelet response to AA was independently associated with intracardiac thrombosis (7). Consistent with our clinical findings, high-dose AA in platelets resulted in a biphasic response, characterized at first by platelet inhibition, which was diminished by LPS-mediated TLR4 signaling. Two plausible mechanisms could explain this unique priming process. First, AA, which is a phospholipid, is catalyzed by cyclooxygenase 1 (COX-1) to produce not only thromboxane A_2_ but also prostacyclin and prostaglandin E_2_. Prostacyclin and prostaglandins elicit inhibitory signals via their respective receptors, EP2 and EP4, to activate the enzyme, adenyl cyclase, converting adenosine triphosphate to cyclic monophosphate (cAMP) (26, 27). Increased intracellular cAMP activates protein kinase A, which phosphorylates a variety of downstream proteins, including P-VASP, and subsequently downregulates signaling pathways and platelet integrin activation (28, 29) (Figure 5). Decreased P-VASP following LPS treatment suggests that TLR4 signaling in feline platelets may result in decrease in intracellular cAMP or cGMP and subsequent modulation of downstream inhibitory signals, resulting in platelet priming (6). One plausible mechanism is that TLR4 activation in feline platelets may activate the enzyme, phosphodiesterase (PDE), responsible for metabolizing and decreasing intracellular cAMP or cGMP. In macrophages, TLR4 activation is mediated by inhibition of PDE4, which induces the downstream signal of cAMP/PKA to upregulate interleukin1 receptors (30). Similarly, platelet inhibition occurs when LPS-treated human platelets were co-treated with the pan-phosphodiesterase inhibitor, IBMX (31). This suggests that cats and humans may share a common cAMP/cGMP dependent pathway of TLR4. Additionally, in human and canine models, LPS-mediated platelet activation has been shown to involve the COX-TxA_2_ axis, and our data suggest that similar mechanisms are also conserved in cats. The ability of LPS to potentiate TxA_2_ production and negate prostaglandin-dependent inhibitory pathways provides a mechanistic explanation for the heightened platelet activity observed in cats with systemic inflammation or cardiovascular disease.

Clinically, these results may have important implications for cats with thromboembolic diseases. In addition to LPS, other TLR4 agonists such as histones, not only are enriched in feline arterial thrombi but also are released into the circulation via neutrophil extracellular traps in HCM-affected cats (15). Rather than directly activating platelets, damage-associated molecular patterns like histones may initiate immunothrombosis by lowering the activation threshold of platelets, and thereby facilitating inappropriate thrombus formation in the presence of secondary agonists. The observation that TLR4 activation reverses AA-mediated platelet inhibition under basal conditions may explain the high on-treatment recurrent thrombosis in cats that had experienced CATE (3). Antiplatelet drugs alone like aspirin or clopidogrel are unlikely to dampen the priming response elicited by TLR4. This underscores the importance of investigating novel therapeutic targets such as targeting TLR4 signaling or its downstream cAMP/cGMP pathway to restore prostaglandin-dependent inhibitory signaling and mitigate platelet-driven hypercoagulability in cats with high thrombotic risk.

This study has several limitations. First, our experiments were conducted exclusively *in vitro*, using platelets from clinically healthy cats. Whether similar mechanisms occur *in vivo*, particularly in cats with cardiomyopathy or systemic inflammation, remains to be understood. Second, our sample size was relatively small, though consistent effects were observed across biological replicates. While we demonstrated that LPS effects were mediated through TLR4, downstream signaling pathways and interactions with other innate immune receptors remain to be elucidated. Finally, we did not delineate the downstream effects of TLR4 by measuring intracellular phosphorylation of protein kinases, cAMP or cGMP directly.

In conclusion, feline platelets express functional TLR4, which reverses arachidonic acid–mediated inhibition by enhancing TxA_2_ production and attenuating prostaglandin-dependent inhibitory pathways. These findings offer novel insights into the immunothrombotic function of platelets in cats. Future studies should characterize platelet TLR4 expression and its signaling in cats at risk of CATE and evaluate potential therapeutic interventions to target platelet-driven immunothrombosis.

## Data Availability

The original contributions presented in the study are included in the article/supplementary material, further inquiries can be directed to the corresponding author.
